# Detection of muscle stem cell-derived myonuclei in murine overloaded muscles

**DOI:** 10.1016/j.xpro.2022.101307

**Published:** 2022-04-11

**Authors:** Akihiro Kaneshige, Takayuki Kaji, Hayato Saito, Tatsuyoshi Higashimoto, Ayasa Nakamura, Tamaki Kurosawa, Madoka Ikemoto-Uezumi, Akiyoshi Uezumi, So-ichiro Fukada

**Affiliations:** 1 Project for Muscle Stem Cell Biology, Graduate School of Pharmaceutical Sciences, Osaka University, 1-6 Yamada-oka, Suita, Osaka 565-0871, Japan; 2 Biological/Pharmacological Research Laboratories, Central Pharmaceutical Research Institute, Japan Tobacco Inc., 1-1 Murasaki-cho, Takatsuki, Osaka 569-1125, Japan; 3 Laboratory of Molecular and Cellular Physiology, Graduate School of Pharmaceutical Sciences, Osaka University, 1-6 Yamada-oka, Suita, Osaka 565-0871, Japan; 4 Department of Nutritional Physiology, Institute of Medical Nutrition, Tokushima University Graduate School, 3-18-15 Kuramoto-cho, Tokushima 770-8503, Japan; 5 Laboratory of Veterinary Pharmacology, Department of Veterinary Medical Sciences, Graduate School of Agriculture and Life Sciences, Tokyo University, 1-1-1 Yayoi, Bunkyo-ku, Tokyo 113-8657, Japan

**Keywords:** Cell Biology, Microscopy, Stem Cells

## Abstract

Muscle satellite cells (MuSCs) supply nuclei to existing myofibers in response to mechanical loading. This myonuclear accretion is critical for efficient muscle hypertrophy. Herein, we present protocols for the detection of MuSC-derived new myonuclei in loaded mouse muscle, including procedures for EdU injection to stain myonuclei, followed by surgery and skeletal muscle fixation. We then describe immunostaining for EdU^+^ myonuclei and image acquisition for quantitative analyses.

For complete details on the use and execution of this protocol, please refer to Kaneshige et al. (2022).

## Highlights

• Surgical procedure for mechanical loading of the plantaris muscle
• Fundamental procedure for preparing frozen muscle samples
• Detailed description of immunostaining for EdU+ myonuclei
• Acquisition of images for quantitative analyses

## Graphical abstract

**Figure:**
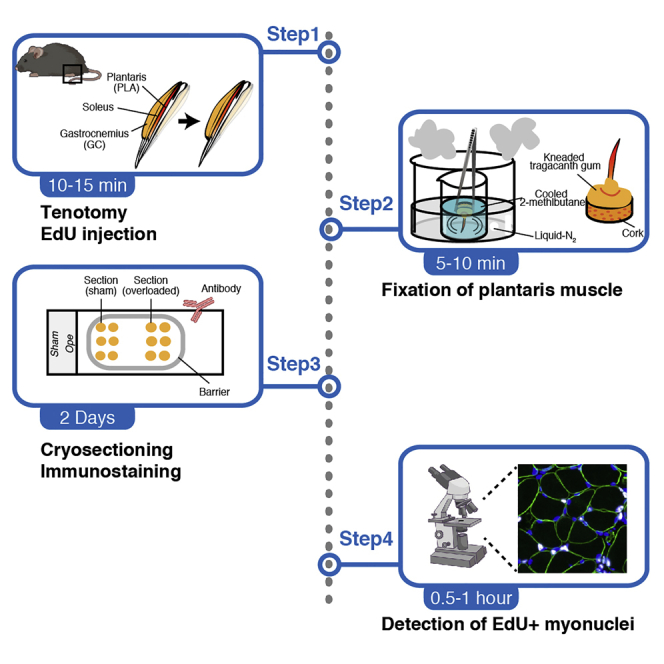

## Before you begin

### Institutional permissions

**Timing: Ask own institute**

1. Before starting this experiment, protocols must be approved by institutional experimental animal care and use committee of own facility.

### Sterilization of instruments for surgical procedures

**Timing: 2 days**

**Figure 1:**
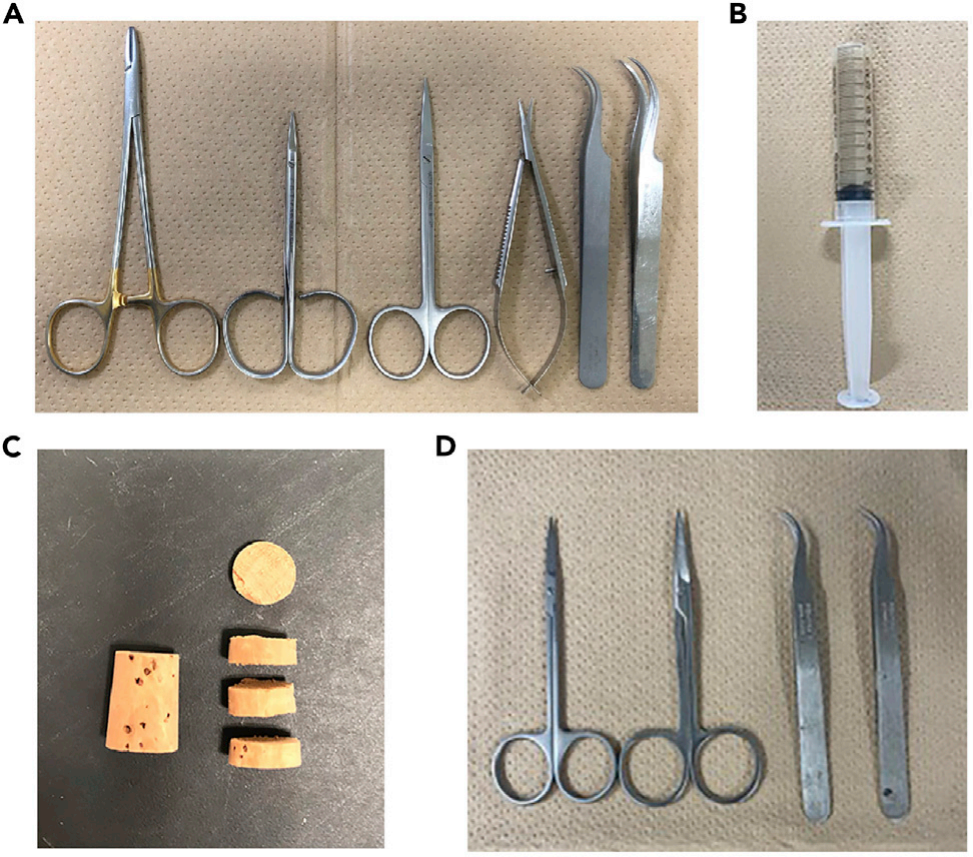
Tools for surgical operation and muscle isolation/fixation (A) Photograph of the surgery equipment needed for tenotomy. (B) Photograph of the kneaded tragacanth gum packed in a syringe. (C) Photos of the original and cut cork. (D) Photograph of the surgery equipment for plantaris muscle resection.

2. Sterilize the surgical instruments (Figure 1A).
  a. Sterilize two scissors, one micro-scissors, two curve-end forceps, and one needle holder in an autoclave (120°C, 20 min).
  b. Dry the autoclaved surgical instruments for 16–24 h.

### Preparation of anesthesia cocktail

**Timing: 5 min**

3. Prepare anesthesia cocktail (see materials and equipment for recipes).

### Preparation of reagents for EdU (5-ethynyl-2′-deoxyuridine) injection

**Timing: 10 min**

4. Prepare the EdU solution.
  a. Dissolve EdU to 2.5 mg/mL in sterilized PBS and store at −20°C.
    ***Note:*** To avoid repeated freeze and thaw, the 2.5 mg/mL EdU stock solution can be stored in 200–500 μL volume aliquots in a microcentrifuge tube. Then, only the amount of diluted solution needed for the day can be prepared. The stocked solution is used within approximately 6 months.
  b. Thaw aliquot and dilute the 2.5 mg/mL EdU stock solution to 0.5 mg/mL with sterilized PBS before administration into mice. Only the amount of 0.5 mg/mL EdU solution needed for the day is prepared.

### Preparation for muscle fixation

**Timing: 2 days**

5. Knead the tragacanth gum.
  a. Knead tragacanth gum powder with H_2_O or PBS until it becomes as hard as an earlobe. Add 10 mL H_2_O or PBS to 5 g tragacanth gum powder while kneading.
  b. Load all kneaded tragacanth gum into a 10 mL syringe with the tip cut off (Figure 1B) and store at 4°C. Approximately 500 mg of the knead tragacanth gum is necessary for the fixation one muscle.

***Note:*** Mold may grow when stored for a long time; therefore, the kneaded tragacanth gum should be used within a month.

6. Cut the cork (Figure 1C).

***Note:*** Soft corks, that change shape when pinched, are not suitable. The height of the cork is also important because it may cause the specimen to move when making frozen sections which break the specimen or hamper making uniform thickness of sections. In addition, the higher cork also will increase risk that the cork will separate from the sample stand when the cryostat blade strikes the sample. Therefore, cut the cork to an appropriate height (approximately 4 mm-height and 11–13 mm ø).

7. Sterilize surgical instruments for dissection (Figure 1D).
  a. Sterilize two scissors and two forceps in an autoclave (120°C, 20 min).
  b. Dry the autoclaved surgical instruments for 16–24 h.

### Preparation for immunostaining

**Timing: 120 min**

8. Dissolve paraformaldehyde (PFA) powder in PBS.
  a. Weigh 0.2 g of PFA and add 5 mL of PBS to create a 4% PFA solution.
  b. Warm the solution at 60°C–70°C.
  c. Mix and vortex every 20–30 min until PFA is completely dissolved. It takes about 3–4 h.
  d. Keep the solution on ice.

***Note:*** PFA is a hazardous chemical. PFA should be handled with adequate ventilation and preferably in a fume hood. This step should be done with gloves on. Only the amount of 4% PFA solution needed for the day is prepared.

***Note:*** 1 mL of 4%PFA is required for one slide.

9. Dissolve skim milk powder in PBS.
  a. Weigh 0.2 g of skim milk and add 4 mL of PBS to create a 5% skim milk solution.
  b. Mix the solution well.
  c. Keep the solution on ice.

***Note:*** To avoid contamination, prepare only the amount needed for the day. At least, 200–300 μL skim milk solution will be necessary for ten sections blocking and staining.

10. Confirm or set the temperature of the cryostat for tissue sectioning.

***Note:*** −25°C is adequate for sectioning of skeletal muscle.

## Key resources table

**Table:** 

REAGENT or RESOURCE	SOURCE	IDENTIFIER
**Antibodies**		

Rabbit Anti-Dystrophin Polyclonal (1:800 dilution)	Abcam	Cat# AB15277; RRID: AB_301813
Donkey anti-Rabbit IgG (H+L) Highly Cross-Adsorbed Secondary Antibody, Alexa Fluor 488 (1:500 dilution)	Thermo Fisher Scientific	Cat# A21206; RRID: AB_2535792

**Chemicals, peptides, and recombinant proteins**		

EdU	Thermo Fisher Scientific	Cat# A10044
2-methylbutane	FUJIFILM Wako Pure Chemical Corporation	Cat# 168-09195
Tragacanth Gum, Powder	FUJIFILM Wako Pure Chemical Corporation	Cat# 200-02245
Paraformaldehyde (PFA)	FUJIFILM Wako Pure Chemical Corporation	Cat# 162-16065
Sucrose	FUJIFILM Wako	Cat# 196-00015
Triton-X100	Sigma-Aldrich or Nacalai Tesque	Cat# T8787 Cat# 35501-15
Pap pen (DAKO pen)	Agilent Technologies	Cat# S2002
Tissue-Tek OCT compound	Sakura-Finetek Japan	Cat# 4583
VECTASHIELD Mounting Medium with DAPI	Vector	Cat# H1200
Skim milk	MORINAGA MILK INDUSTRY CO., LTD	Cat# 0652842
Isoflurane	Pfizer	Cat# 4987-114-13340-3
Medetomidine (Domitor)	ZENOAQ	Cat# WAK0001
Midazolam (Dormicum Injection 10 mg)	Astellas	Cat# 4987-211-76210-0
Butorphanol (Vetorphale)	Meiji Seika Pharma	Cat# WAK-52850
Ampicillin sodium salt	Sigma-Aldrich	Cat# A9518-5G

**Critical commercial assays**		

Click-iT(TM) EdU Alexa Fluor™ 647 Imaging Kit	Thermo Fisher Scientific	Cat# C10340

**Experimental models: Organisms/strains**		

Mouse: C57BL/6J (10–15 weeks, male or female)	Charles River Laboratories	N/A
Mouse: *B6;129-Pax7tm2.1(cre/ERT2)Fan/J* (10–15 weeks, male or female)	The Jackson Laboratory	JAX:# 012476; RRID:IMSR_JAX:012476
Mouse: *B6N.Cg-Tg(Pdgfra-cre/ERT)467Dbe/J* (10–15 weeks, male or female)	The Jackson Laboratory	JAX:# 018280; RRID:IMSR_JAX:018280

**Software and algorithms**		

Hybrid Cell count	Keyence	https://www.keyence.com/search/all/?q=hybrid+cell+count
BZ-X Analyzer	Keyence	https://www.keyence.com/search/all/?q=BZ-X%20analyzer&o=0

**Other**		

All-in-one Fluorescence Microscope BZ-X700	Keyence	N/A
Scissors	Fine Science Tools	Cat# 14040-10
Scissors	Napox	Cat# B-12H
Needle holder	Fine Science Tools	Cat# 13014-14
Needle holder	Napox	Cat# C-36-T1
Forceps	FONTAX	Cat# No7
Micro-scissors	Napox	Cat# MB-50-10
Suture with Needle (E.O.G Stealized)	Natume	Cat# CA1360N1NT
Storage container, 20 mL Screw Bottle	Maruemu Corporation	Cat# 4-1024-04
10 mL Syringe	Terumo	Cat# SS-10SZ
Hair Clipper (Pet Club Dog Clippers for Partial Cut Blue Set of 5)	Panasonic	Cat# ER803PP-A
Silane coated slide glass	Muto pure chemical co.LTD	Cat# 511618
Cover glass	Matsunami	Cat# C024601
Cork	SANSYO	Cat# 94-0981
Conical beaker 100 mL	Iwaki	Cat# 82-0032
STABLO-AP	Shimazu	Cat# 321-73700-01
Filter cap	Natsume Seisakusyo	Cat# KN-617
NARCOBIT-E	Natsume Seisakusyo	Cat# KN-1017
15 mL tube	Corning	Cat#430791

## Materials and equipment

**Table:** Anesthesia cocktail

Reagent	Final concentration	Amount
Medetomidine (1 mg/mL)	0.03 mg/mL	0.75 mL
Midazolam (5 mg/mL)	0.4 mg/mL	2 mL
Butorphanol (5 mg/mL)	0.5 mg/mL	2.5 mL
Sterilized water		19.75 mL
**Total**	**n/a**	**25 mL**

Store the anesthesia cocktail at 15°C–25°C up to 3–6 months.

***Alternatives:*** Isoflurane with device (e.g., NARCOBIT-E) is also acceptable.

**Table:** Surgical equipment A (for tenotomy)

Material	Amount
Needle holder	1
Scissors	2
Micro-scissors	1
Curve-end forceps	2

**CRITICAL:** Use scissors that fit the size of your hand and are easy to handle. All materials must be sterilized. To avoid infection, separate scissors and forceps used for cutting skin from those used for cutting tendons. An example is shown in Figure 1A.

**Table:** Surgical equipment B (for dissection)

Material	Amount
Scissors	2
Curve-end forceps	2

**CRITICAL:** Use scissors that fit the size of your hand and are easy to handle. All materials must be sterilized. An example is shown in Figure 1D.

## Step-by-step method details

### Tenotomy and EdU injection

**Timing: 10 min per muscle**

This step describes the surgical procedure for mechanical loading of the plantaris muscle by cutting the tendon of the gastrocnemius and soleus muscles. Before starting this experiment, protocols must be approved by institutional experimental animal care and use committee of own facility.

**Figure 2:**
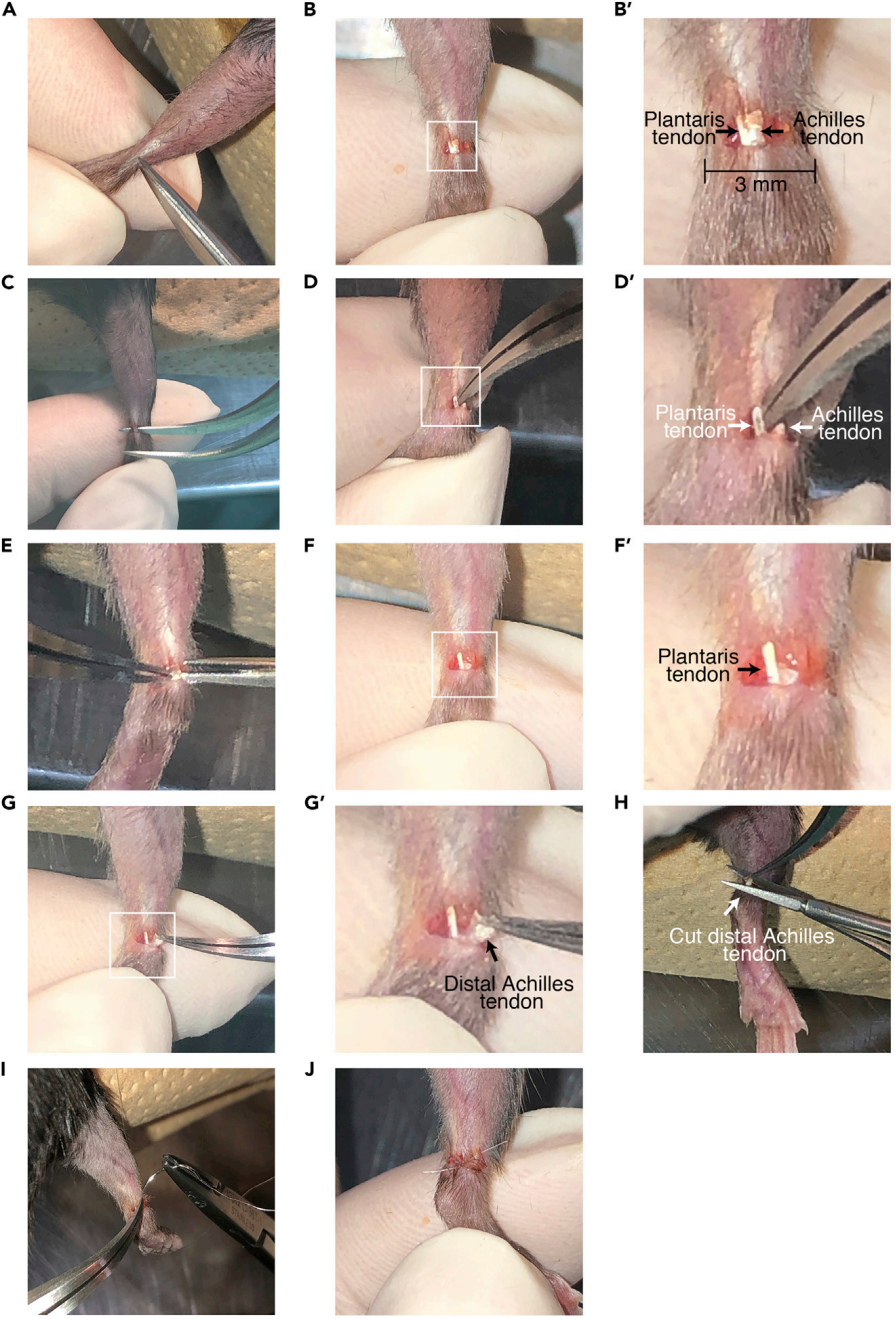
Procedure for cutting the tendon of gastrocnemius and soleus muscles (A) Picture showing the effect of anesthesia by stimulating the sole of the foot and the incision position. (B) Picture of cut skin. After cutting the skin, two distinct tendons appear. The incision was made larger to clearly show the tendon. (C) Picture showing insert the tips of the forceps into the underside of both tendons for cutting gastrocnemius and soleus tendons (Achilles tendon). (D and E) Pictures of the cutting of Achilles tendon. Use the tip of the forceps, lift the gastrocnemius and soleus tendons to create a space for the micro-scissors to be inserted, and then precisely cut through the tendons. (F) Picture of plantaris tendon after cutting Achilles tendon. (G and H) Pictures showing removal 1 mm of Achilles tendon. (I) Picture of the suturing process. (J) Picture of the end of surgery. (B′, D′, F′, and G′) Magnified images of the area enclosed by the squares in (B), (C), (F,) and (G).
3. Sanitize and wipe skin with 70% alcohol or related reagents (e.g., 10% povidone-iodine).
4. Cut the skin to expose the plantaris and Achilles tendon (Figures 2A and 2B).

***Note:*** The incision position is approximately 3–5 mm from the heel. The risk of damage to the plantaris tendon may increase if the incision is too proximal. As tendon exists just beneath the skin, carefully cut the skin. The width of incision should be approximately 2–3 mm.

1. Anesthetize adult mice (e.g., C57BL/6J, male or female, 10–15 weeks) by Isoflurane with device (e.g., NARCOBIT-E) or anesthesia cocktail (80–100 μL per 10 g) are used. Both male and female aged 10–15 weeks are used.
2. Shave the fur from the hind limbs using hair clippers (Figure 2A).

5. Transect the distal Achilles tendons of both gastrocnemius and soleus muscles (Figures 2C–2F).

**CRITICAL:** Special care must be taken not to damage the plantaris tendon and vasculature around the Achilles tendon.

6. Remove 1 mm of the tendon to avoid reconnection of the cut tendon during the experimental time course (Figures 2G and 2H).
7. Suture the skin using surgical needle (Suture with Needle (E.O.G Stealized)) with suture and a needle holder (Figures 2I and 2J).

***Optional:*** When the contralateral muscle is used as a sham control, skip steps 5 and 6.

***Note:*** There are several types of suture; the materials and size. Non-absorbable nylon and 6/0 size suture is used in our surgical procedure. The needle is a square type for plastic surgery.

8. Inject 0.5 mg/mL EdU dissolved in PBS intraperitoneally into mice at 5 mg/kg body weight daily until the day before euthanizing.

***Note:*** 4 days after tenotomy is too short to detect EdU-positive myonuclei. Samples 7 days after tenotomy were used in our studies.

### Fixation of the plantaris muscle

**Timing: 2–5 min per muscle**

This is a basic procedure for preparing frozen muscle samples.

9. Dissect the plantaris muscle with sterile scissors and forceps.

**Figure 3:**
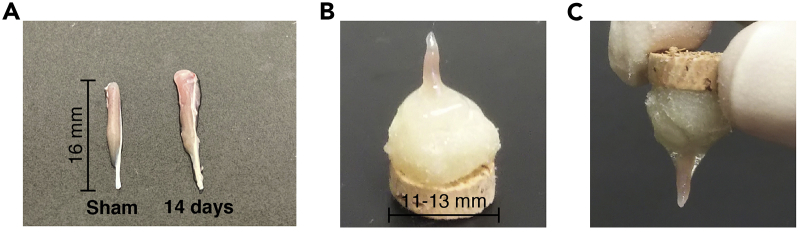
Preparation of plantaris muscle for rapid freezing (A) Pictures of plantaris muscle from contralateral sham (left) or overloaded muscle 14 days after tenotomy (right). (B) Pictures of plantaris muscle standing on kneaded tragacanth gum. (C) Pictures of plantaris muscle upside down.

***Note:*** An apparent increase in the size of the plantaris muscle can be observed compared to that of the contralateral sham control (Figure 3A). Loaded muscle weight on day 7 after tendon rupture was increased by approximately 20%–40% compared to the contralateral sham control.

***Note:*** Used dissection tools are sterilized after washing.

**Figure 4:**
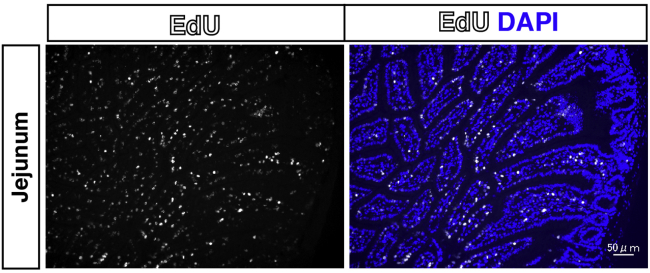
Positive control for EdU staining Immunostaining of EdU (white) in jejunum from EdU-treated operated mice. Nuclei were counterstained with DAPI. Scale bar: 50 μm.

***Optional:*** For a positive control of EdU staining, we recommend isolating the jejunum and fixing it using the same protocol as for muscle fixation (Figure 4).

***Optional:*** If the target is a soluble factor, including fluorescent proteins, the isolated plantaris muscle must be fixed with 4% PFA for 30 min, followed by sucrose replacement (10% sucrose/PBS 4°C for 3 h, then in 20% sucrose/PBS 4°C for 12–16 h). 10 mL 4% PFA and 15–20 mL each percentage of sucrose dissolved PBS is necessary for one muscle. Storage container, 20 mL Screw Bottle, or 15 mL tube is used for this step. After sucrose replacement, you can continue from step 10. Sucrose solutions are used on the same day.

10. Pick the distal tendon of the isolated plantaris muscle with forceps and place it on the cork using kneaded tragacanth gum (Figure 3B). Fill the space between the muscle and gum with Tissue-Tek OCT compound.

***Optional:*** If the sample does not stand up straight, turn it upside down and soak it in cooled 2-methylbutane (Figure 3C). At this time, one should be careful not to let the sample touch the bottom or the wall of conical beaker.

11. Freeze the plantaris muscle in liquid nitrogen-cooled 2-methylbutane in conical beaker for 1 min with vigorous shaking by holding the cork with a pair of forceps.

**Figure 5:**
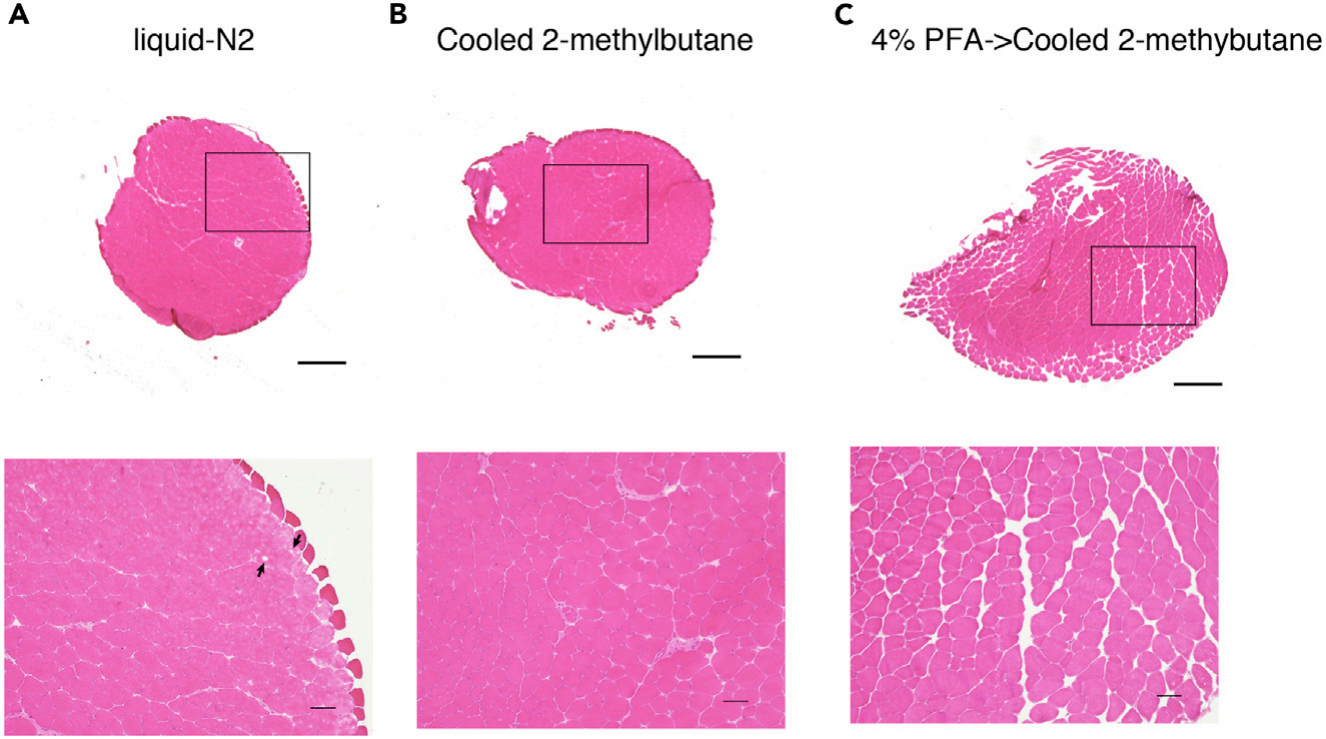
Rapid freezing is essential for muscle fixation (A–C) H&E staining of plantaris muscles frozen in liquid nitrogen (A), liquid nitrogen-cooled 2-methylbutane (B), or liquid nitrogen-cooled 2-methylbutane after 4% PFA fixation (C). In (A), uneven staining and holes (arrows) are observed in the myofibers. In (C), myofiber divergence is observed, which may affect the volume of interstitial spaces. Scale bar: 200 μm.

**CRITICAL:** Liquid nitrogen-cooled 2-methylbutane and vigorous shaking are essential for freezing the sample as rapidly as possible. If this process is insufficient or the samples are directly placed into liquid nitrogen, a hole(s) and mottled staining will be seen in the myofibers (Figure 5).

***Note:*** Wait until the 2-methylbutane cools down completely and the white vapor disappears. Then, immerse the muscle sample into the 2-methylbutane cooling with liquid nitrogen. If 2-methylbutane is immersed in liquid nitrogen for a long time, it will freeze; therefore, 2-methylbutane should be removed from liquid nitrogen to allow the frozen 2-methylbutane to melt. Again, the melted 2-methylbutane is put into liquid nitrogen and wait until the white vapor disappears, then immerse the muscle sample into the 2-methylbutane.

12. Place the muscle samples on dry ice for 1 h to vaporize the 2-methylbutane.
13. Store the muscle samples in closed containers (e.g., 20 mL Screw Bottle) at −80°C.

***Note:*** Place the sample in a sealable container and avoid grasping the container by hand to prevent thawing of the sample. The plantaris muscle may be damaged by physical shock (e.g., dropping samples or containers while transport) and should be handled with care (Do not shake and drop the container and samples).

### Immunostaining

**Timing: 2 days**

This step describes immunostaining for EdU^+^ myonuclei.

14. Make transverse cryosections (6 μm thick) of the plantaris muscles using a cryostat.
  a. Take samples stocked in containers from the –80°C freezer and place in a cryostat.
  b. Keep the samples in the cryostat for 10 min.
  c. Make transverse cryosections (6 μm thick) on silane coated slide glass.

***Note:*** When stocked samples in −80°C deep freezer are not placed next to the cryostat, please transport samples on dry ice, but not in liquid-nitrogen.

***Note:*** After cutting off at least 1 mm of the proximal side of the plantaris muscle, cut non-sequential 10 cryosections per one muscle. Totally, 40 cryosections can be placed on one slide.

15. Dry and make a hydrophobic barrier around the section.
  a. Dry slides for 30 min at 15°C–25°C to ensure that cryosections completely attach on slide glass.
  b. After confirming that the glass slide is not wet, trace around cryosections two or three times with a PAP pen (DAKO pen) to create a barrier within 30 min.
  c. Dry the barrier completely for 5 min at 15°C–25°C.

***Note:*** 30 min is a minimum time, several hours is also acceptable. Do not push the PAP pen tip too strongly onto the slide because excessive liquid leakage will occur and ruin the sample.

16. Fixation.
  a. Cover the section with 4% PFA dissolved in PBS (approximately 1 mL/slide) and incubate it for 10 min at 15°C–25°C.
  b. Place the slides in a slide staining jar (80–100 mL) and wash three times with 1× PBS containing 0.1% Triton X.

***Note:*** Aqueous solutions containing PFA should not be disposed of in the sink but should be properly disposed of as hazardous waste.

17. Permeabilization.
  a. Treat the slides with 1× PBS containing 0.5% Triton X (approximately 0.5 mL/slide) and incubate for 20 min at 15°C–25°C.
  b. Place the slides in a slide staining jar and wash thrice with 1 × PBS.
18. EdU staining.
  a. Prepare a reaction cocktail of the Click-iT® EdU Imaging Kit (Click-iT (TM) EdU Alexa Fluor™ 647 Imaging Kit) according to the manufacturer’s instructions.
  b. Cover the sections with reaction cocktail (approximately 0.5 mL/slide) and incubate for 30 min in the dark at 15°C–25°C.
  c. Place the slides in a slide staining jar and wash thrice with 80–100 mL 1× PBS containing 0.1% Triton X.
19. Blocking.
  a. Cover the sections with 5% skim milk dissolved in PBS (approximately 0.5 mL/slide) and incubate for 1 h in the dark at 15°C–25°C.
20. Primary antibody treatment.
  a. Prepare the primary antibody against dystrophin protein (rabbit anti-dystrophin polyclonal antibody) by diluting 800 times with 5% skim milk dissolved in PBS (approximately 0.5 mL/slide).
  b. Cover the sections with diluted primary antibody and incubate in humidified chamber in the dark at 4°C for 12–16 h.
  c. Place the slides in a slide staining jar and wash thrice with 80–100 mL 1× PBS containing 0.1% Triton X.

***Note:*** Only the amount of the primary antibody solution needed for the day is prepared. Do not sore the diluted antibody.

21. Secondary antibody treatment.
  a. Prepare the secondary antibody (donkey anti-rabbit antibody, Alexa Fluor 488) by diluting 500 times with 1× PBS (approximately 0.5 mL/slide).
  b. Cover the sections with diluted secondary antibody and incubate for 1 h in dark at 15°C–25°C.
  c. Place the slides in a slide staining jar and wash thrice with 80–100 mL 1× PBS containing 0.1% Triton X.

***Note:*** Only the amount of the secondary antibody solution needed for the day is prepared. Do not sore the diluted antibody.

22. Mounting.
  a. Cover the sections with VECTASHIELD® Mounting Medium containing DAPI (3–4 drops/slide).
  b. Lay cover glass on the sections.
  c. Place tissue paper on the slides and wipe the excess medium.
  d. Fix the cover glass with nail polish.

### Detection of EdU^+^ myonuclei

**Timing: 0.5–1 h per section**

This is the step for acquiring images for quantitative analyses.

23. Adjust the exposure time and gain of each channel to blue (DAPI), green (dystrophin), and white (EdU).

***Note:*** Dystrophin staining might be better than EdU staining for adjusting the focus. To avoid the loss of fluorescent signals, images should be acquired on the same day.

***Note:*** Low-resolution images make it difficult to determine the location of EdU. A high-magnification lens (x20 objective lens) should be used for the subsequent analyses.

24. Acquire individual blue, green, and white images in all areas of the plantaris muscle using All-in-one Fluorescence Microscope BZ-X700.

**Pausepoint:** Confirm the saved data and shut down the PC.

**Figure 6:**
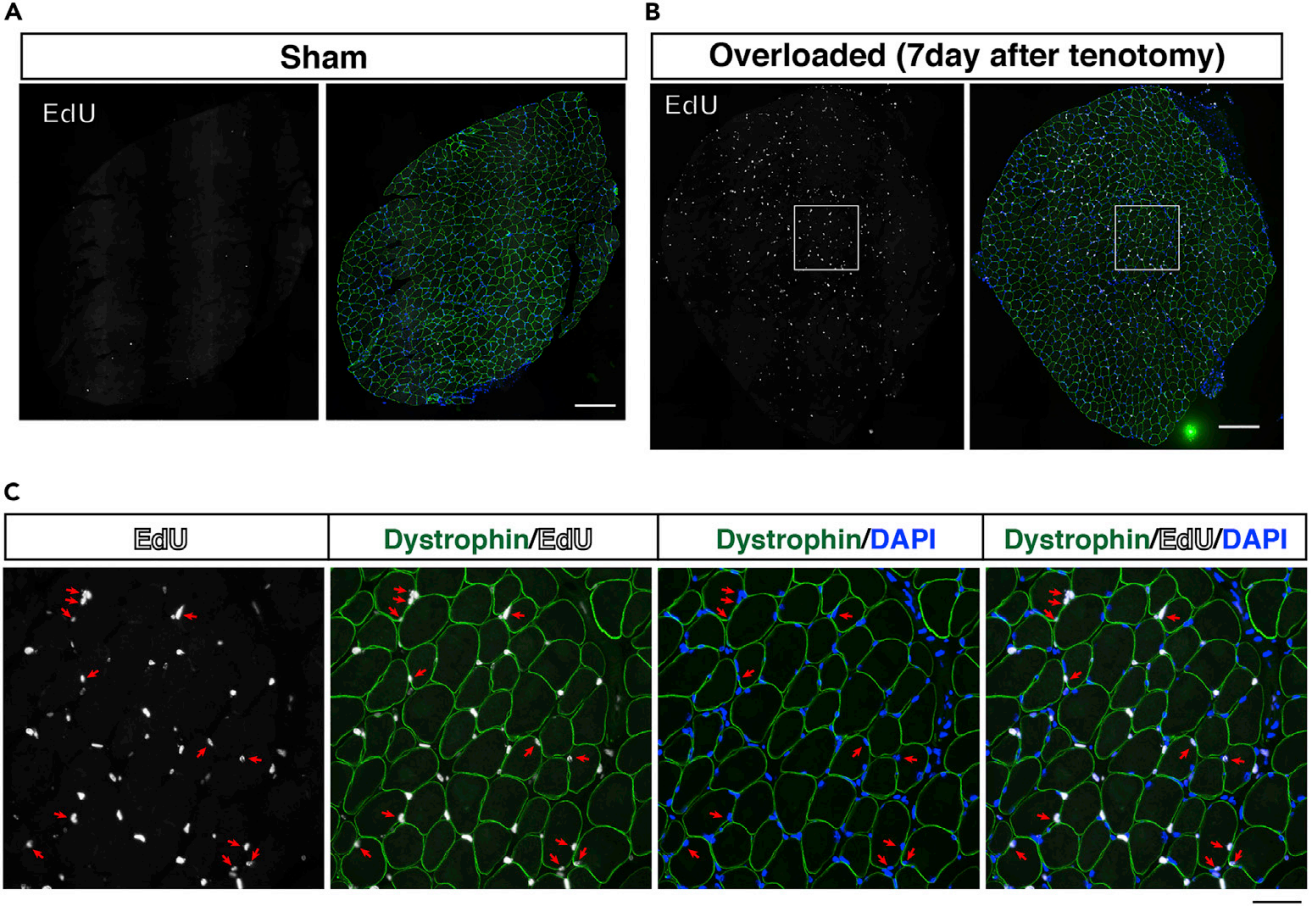
Detection of EdU^+^ myonuclei in a section (A and B) Immunostaining of dystrophin (green) and EdU (white) in Sham (A) and overloaded (B) plantaris muscles on day 7 after tenotomy. Scale bar: 200 μm. (C) Magnified images of the area enclosed by the squares in B. EdU^+^ nuclei located within the dystrophin marked myofibers (arrows) are defined as new myonuclei derived from muscle stem cells. Scale bar: 50 μm.

***Note:*** If there is a sample for which it is difficult to acquire a complete and clean image, the number of myofibers can be counted and the number of EdU^+^ myofiber nuclei per 100 myofibers can be calculated. In this case, the data are presented as EdU^+^ number per 100 myofibers.

25. Use Keyence soft system (BZ-X Analyzer and Hybrid Cell count) or similar system to generate stitched images.
26. Count the number of EdU^+^ myonuclei located within myofibers marked by the dystrophin staining. DAPI staining is essential for confirming the myonuclei (Figure 6).

## Expected outcomes

Before quantification, the increased muscle weight allowed us to determine a successful surgical operation. Using C57BL/6 mice, the plantaris muscle weight increased about 20%–40% compared to that of the sham muscle on day 7 after surgery.

This method allows us to detect and quantify EdU^+^ myonuclei because existing myonuclei never uptake EdU and nuclei of other types of cell including MuSC are located outside of dystrophin staining. For example, we found approximately 70 ± 17 (SD) EdU^+^ myonuclei per section on day 7 after tenotomy in the control of *Pdgfra*^*CreERT*^ and 24 ± 12 EdU^+^ myonuclei per section were detected in mesenchymal progenitors-depletion mice (*Pdgfra*^*CreER*^^*T*^*::Rosa*^*DTA*^) 7 days after tenotomy (Kaneshige et al., 2022). In normal C57BL/6 mice, approximately 6 ± 1.8 and 15 ± 5.5 EdU^+^ myonuclei per 100 myofibers in the overloaded plantaris muscle on days 7 and 14, respectively (Fukuda et al., 2019). Per section, approximately 60–100 EdU^+^ myonuclei were detected on day 7 after tenotomy in C57BL/6. In our analysis, similar results were obtained from male and female mice. In sham muscle, EdU^+^ myonuclei are barely detected. But if young mice (8–10 weeks) are used, several EdU^+^ myonuclei in sham muscle might be detected as their muscle are still growing.

## Limitations

### Data variation

The major limitation of immunohistochemical staining of the sections is that only a part of the sample is observed. In addition, there is large variation in the data of the same group as the number of EdU^+^ myonuclei depends on the degree of muscle loading by the activity level of mouse. We recommend analyzing at least three non-serial sections per sample and calculating the average number of EdU^+^ myonuclei per mouse. We also recommend to use sections after cutting off at least 1 mm of the proximal side of the plantaris muscle and compare the sections of approximately the same distance from the proximal side of the muscle.

### Mild and attenuated muscle growth

Muscle growth by tenotomy is mild and stops 2–3 weeks after surgery. Therefore, the tenotomy model is not suitable for the analyses of the late stage of muscle hypertrophy (Fry et al., 2014; Fukada et al., 2020). In contrast, the tenotomy model minimizes myofiber damage compared to synergistic ablation (McCarthy et al., 2011; Murach et al., 2017). Therefore, this model is suitable for early events of muscle response to increased mechanical loads. Especially, the tenotomy model is suitable for analyses of MuSCs behaviors in loading muscle as the experiments can be performed without the effects of degeneration and regeneration processes.

## Troubleshooting

### Problem 1

Infection or death in operated mice (after the surgical operation in steps 1–7).

### Potential solution

If mice are kept in conventional animal facility, the operated mice might be infected and die. To reduce this risk, the incision should be made as small as possible and consider to use disinfectant (10% povidone-iodine). In addition, the operated mice are kept in a cage with clean animal bedding, and the cage is covered by a filter cap (e.g., Filter cap, Natsume Seisakusho). Antibiotic, e.g., ampicillin (drinking water, 1 g/L) given to mice will reduce the risk of infection.

### Problem 2

Plantaris muscle weight is not increased compared to that of the contralateral sham control. (step 9 in the dissection of plantaris muscle).

### Potential solution

The plantaris muscle is not loaded with sufficient mechanical cues for several reasons. One is the incomplete resection of the soleus and gastrocnemius tendons. You will notice this failure when the mice are sacrificed. The second possibility is the unsuccessful removal of the plantaris muscle, as a loaded plantaris muscle sometimes adheres to the gastrocnemius muscle, and the cut gastrocnemius tendon adheres to the plantaris muscle. The anatomical positional relationship between the plantaris and gastrocnemius muscles should be determined using sham or non-loaded muscles. The loss of muscle or attachment of other muscles or tissues in sections can be confirmed. The third possibility is the damage to the plantaris tendon, which do not allow the plantaris muscle to receive a sufficient mechanical loading. This is also confirmed when the mice are sacrificed. The fourth possibility is the low activity of the mice. The operated mice should not be kept in small cages for single animals (136 × 208 × 115 mm). We use 225 × 338 × 140 mm sized cages.

### Problem 3

Attaching sections to a glass slide is critical to obtain entire images. Arranging the sections neatly is also important to save the volume of antibody and to distinguish the sample easily. (Making transverse section on slide glass in step 14).

### Potential solution

A considerable factor influences the attaching sections to a glass slide is static electricity. In order to remove static electricity, we recommend to put the glass side on wet wipes or paper after confirming that side with section is up. Alternatively, use static eliminator (e.g., STABLO-AP, Shimazu).

### Problem 4

Unsuccessful or uneven staining results in an incorrect number of EdU^+^ myonuclei. Clear staining is essential for counting the number of EdU^+^ myonuclei. (Staining results in step 24).

### Potential solution

Considerable factors influence the quality of immunostaining.

• For section quality, rapid fixation of the isolated plantaris muscle is critical. Never melt the plantaris muscle after frozen fixation.
• Uniform thickness of the cryosection is critical. When creating a section using a cryostat, the handle should be rotated at a constant speed. Unsuccessful or uneven staining in the same part of the cryosection results from poor quality of the frozen sample or the cryosection.
• It might be better to shake sections slowly during the reaction with antibodies.
• Never dry the sample during the immunostaining procedure.
• Always keep the sections away from light after reaction with the EdU cocktail and secondary antibody conjugated with a fluorescent probe.

### Problem 5

Low number of EdU^+^ myonuclei (Result of counted EdU^+^ myonuclei in step 26).

### Potential solution

Confirm each step.

• Rate of increased muscle weight.
• Staining of positive control (jejunum).
• Quality of sample section.

Deficient of mechanical loading or weak-staining results in the low number of EdU^+^ myonuclei. In addition to these technical problems, there is a possibility that your control mice have low EdU^+^ myonuclei as expected. For example, genetic background or transgene might affect the number of EdU^+^ myonuclei. In our case, mice having *CreERT2* gene in *Pax7* locus (Lepper et al., 2009) showed reduced number of EdU^+^ myonuclei compared to C57BL/6 mice. In addition to the result of EdU^+^ myonuclear number, it is important to conduct other experiments, such as the measurement of the number of MuSCs, and for your conclusion.

## Resource availability

### Lead contact

Further requests for information, resources, and reagents should be directed to and will be fulfilled by lead contact So-ichiro Fukada (fukada@phs.osaka-u.ac.jp).

### Materials availability

This study did not generate new unique reagents.

## Data Availability

This study did not generate datasets.
